# Self-medication practices and their characteristics among Iranian university students

**DOI:** 10.1186/s40360-022-00602-5

**Published:** 2022-08-08

**Authors:** Rohaneh Rahimisadegh, Nader Sharifi, Vahid Kohpeima Jahromi, Razieh Zahedi, Zahra Rostayee, Razieh Asadi

**Affiliations:** 1 Health Services Management Research Center, Institute for Futures Studies in Health, Kerman University of Medical Sciences, Kerman, Iran; 2 Department of Public Health, Khomein University of Medical Sciences, Khomein, Iran; 3 Research Center for Social Determinants of Health, Jahrom University of Medical Sciences, Jahrom, Iran

**Keywords:** Self-medication, Students, Rational use of drug, Iran

## Abstract

**Background:**

Self-medication in students, as educated people, is one of the most important health issues. It is known that inappropriate self-medication is harmful for individuals as it has potential risks, disrupts the drug market and increases the per capita financial drug consumption. Thus, the aim of this study is to investigate the habits related to drug use and the prevalence of self-medication practices among university students.

**Methods:**

This cross-sectional study was conducted in 2021 at Jahrom universities/Iran, using an electronic “self-medication practices and medication habits” questionnaire. A total of 848 students from four healthcare and non-healthcare universities participated in the study.

**Results:**

The prevalence of self-medication among students was 44.8%. The most common medicines used for self-medication included cold remedies and sedatives that were used by 33% of the students. About 47% of students stated that they have used antibiotics without a physician’s prescription. The internet and social networks were the source of information on self-medication in about 40% of students. There was no significant difference in self-medication between medical and non-medical students (OR = 0.865, 95% CI: 0.659–1.134). Self-medication in students with underlying disease was higher than students without underlying disease (OR = 2.8, 95% CI: 1.39–5.60).

**Conclusions:**

Self-medication had a high prevalence among students. The best strategy to reduce or eliminate self-medication is to raise students’ awareness and knowledge about potential risk of self-medication practices.

## Introduction

Medication is one of the basic needs of general public and a strategic commodity in all countries. Significant advances made in the field of medication have paved the way for more people to have access to medication, the consequence of which has been the formation of a harmful social phenomenon called irrational use of drugs. Common types of irrational use of drugs include inappropriate use of antibiotics, overuse of injection when the oral drug would be more appropriate, overuse of medication per prescription, prescription of medication regardless of clinical guidelines, failure to follow medication regimen, and self-medication [1]. Self-medication is the first behavior of patients when faced with the early symptoms of diseases. It is also a common health problem. Self-medication and related habits can cause a number of side effects, including intoxication and bacterial resistance, drug dependency, and obscuring the symptoms of a serious illness [2, 3]. Self-medication disrupts the drug market and increases the per capita drug consumption, which put the country’s’ economy under pressure.

Today, self-medication has increased in the world, and its prevalence is higher in developing countries [4]. Previous studies have reported the prevalence of irrational drug use to be about 87% in India [5], 86.4% in Brazil [6], and 55% in Egypt [7]. The prevalence of self-medication in Iran is also not desirable (83.3%), [8]. According to the World Health Organization, Iran is the second largest country in Asia after China and is among the top 20 countries in the world in terms of drug consumption [9]. Therefore, self-medication has become one of the problems in the Iranian health sector.

Self-medication in students, as educated people in the community, is one of the most important health issues [10]. They are considered as role models for other people in terms of health behaviors. Because of their active role in the media and cyberspace, university students can play a more important role in this area. On the other hand, university students are more vulnerable and prone to self-medication due to their social status, greater contact with people in the community, and their responsibilities as future parents [11–13]. Previous studies have revealed that, the prevalence of self-medication practice seemed to be high among students all over the world. In a study, Okyay and Erdoğ revealed that the prevalence of self-medication in students was 63.4% and the most common drugs used by students without prescription were analgesics, antibiotics and cold remedies [14]. A study conducted by Gupta et al. [15] showed that the prevalence of self-medication among students was 78%, and also fever and headache were the most common diseases that were treated with self-medication. Scientific studies have shown that the prevalence of self-medication in Iran is between 18 and 87% [16–19]. On the other hand, many studies have shown that people with higher levels of education have more drug use habits than less educated people [17, 18]. Therefore, the aim of this study is to investigate the habits related to drug use and the prevalence of self-medication practices among university students in Jahrom, Iran.

## Methods

### Study design and setting

This cross-sectional study was conducted in Jahrom, a city in southeast of Iran to evaluate the drug use habits of university students. Data were collected by an electronic researcher-made questionnaire called;

“self-medication practices and medication habits”. It had 24 questions. 23 questions were about self-medication practices and 1 question about source of information in self-medication. The scaled measured for some questions were two options by “Yes” and “No” and for the questions such as” The number boxes of drugs they have unused or unfinished in their house” were five options “zero, 1-3, 4-7, 8-10 and 11 or more”. The score in the questions with two options were between 0 to 1 and for five options were 0 to 4. The questionnaire was designed according to the previous studies. Cronbach’s alpha was used to calculate the internal consistency of questions. In general, Cronbach’s alpha of 0.9 is taken as indication of good internal consistency. The face and content validity of the questionnaire was approved by six specialists. The study protocol was confirmed by Scientific Researches Ethics Committee of Jahrom University of Medical Science (ID number: IR.JUMS.REC.1399.004). Informed consents were obtained from the students and they were told that participation in the study was voluntary.

### Participants

This study was conducted on students from two public and two private universities, one of which was medical and three were non-medical.

The base sample size was calculated as 658 participants. Considering the prevalence use of antiacid use without physicians’ prescription [19] were 11.8 and 20.3% in order among men and women with 20% type II error and 5% margin of type I error and we added 20% for intra-cluster correlation the final sample size were 790 cases. Guest students, non-Iranian students and students who did not wish to participate in the study were excluded from the study.

### Data collection and measurement

After received the ethical code and made the necessary arrangements with the education department in each university; the mobile phone number of students were gotten. The electronic questionnaire sent to students via social media. Students were recruited from all departments by proportional to size approach. Data collection process took about five months (from January to May 2021).

SPSS software was used for data analysis (SPSS, Chicago, IL, USA). Descriptive statistics were presented as frequencies and percentages in tables. Data were analyzed with Pearson, Chi-square and Logistic regression analyses. Logistic regression used to determine the effective factors on self- medication among students. In the first step the crude Logistic regression run, then the variables with *p* value less than 0.2 interred in the adjusted Logistic regression model.

## Results

A total of 848 university students participated in this study from whom, 65.2% were females. The majority of students had a moderate socio-economic status (58.7%), and 86.7% of them were single. The minimum and maximum age of students were 19 and 46 years (Mean ± SD, 22 ± 2.3 years.), and 4.5% (*n* = 38) of them had a chronic disease (Table 1).

**Table 1:** Socio-demographic characteristics of university students

Variable		Frequency	Percent
Sex	Female	553	65.2
	Male	295	34.8
Marital Status	Single	735	86.7
	Married	105	12.4
	Divorcee or widow	8	0.9
University	Medical University	407	48
	Jahrom University	213	25.1
	Payame Noor University	40	4.7
	Azad University	188	22.2
Economic Status	Very Bad	25	2.9
	Bad	68	8
	Moderate	498	58.7
	Good	236	27.8
	Very Good	21	2.5
	Have Chronic Disease	38	4.5

**Fig. 1:**
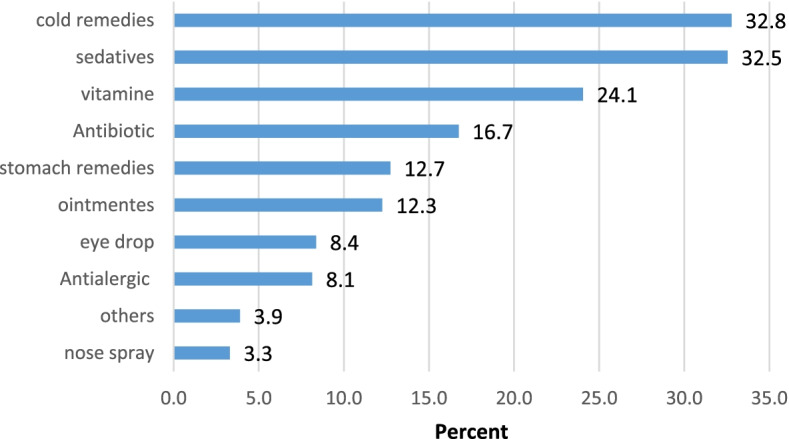
Prevalence of university students with self-medication, according to the medicine used

The prevalence of self-medication among students was 44.8% (*n* = 380). The most common medications used for self-medication were cold remedies and sedatives that were used by about 33% of them (Fig. 1). Also, 27% of students stated that they buy medication from pharmacy without any illness to keep at home. About 8% (*n* = 65) of students declared that they finish the course of treatment, and 31.3% (*n* = 265) of them said that they stop taking the drug after consulting with a physician or a pharmacist. Moreover, 40.7% (*n* = 345) of students stated that they have 1–5 packages of unused or unfinished medication at home and about one-third of them (35.2%) said that had discard about 1–3 packages of medication per year due to expiry date (Table 2).

**Table 2:** Self-reported attitudes of the university students towards drug use

Questions		N	%
Do you use others’ medicines or buy medicines from pharmacy without prescription?	Yes	380	44.8
	NO	468	55.2
	Total	848	100
Do you have medicines prescribed without being sick or buy and keep at home in case of need?	Yes	228	26.9
	NO	620	73.1
	Total	848	100
Have you taken any antibiotics in the last 12 months?	Yes	581	68.5
	NO	267	31.5
	Total	848	100
Do you use antibiotics on your own without a physician’s examination?	Yes	400	47.2
	NO	448	52.8
	Total	848	100
How long do you use the antibiotics prescribed for you?	I quit after the symptoms disappear or a few days after I feel recovered	146	17.2
	I quit at the end of treatment	65	7.7
	I quit after consulting a physician/pharmacist	265	31.3
	I quit a few days after disappear the symptoms	63	7.4
	I quit a few days after whether I feel recovered	198	23.3
	I quit a few days after whether I feel recovered or not	83	9.8
	Others	28	3.3
	Total	146	17.2
How many boxes of drugs do you have unused or unfinished in your house?	None	137	16.2
	1_5	345	40.7
	6_10	116	13.7
	More than 10	250	29.5
	Total	848	100
Over a year, how many boxes of drugs are thrown away even without opening the box, since the expiry date has lapsed?	1_3	300	35.4
	4_7	120	14.2
	8_10	50	5.9
	More than 10	76	9
	None	302	35.6
	Total	848	100
Do you read/check the instructions in the prospectus of the medications you are using?	Yes, always	277	32.7
	Yes, sometimes	496	58.5
	No, I do not	75	8.8
	Total	848	100
How much do you understand about the information in the prospectus of the drug you are using	I understand fully	441	52
	I partially understand	385	45.4
	Questions	N	%
	I understand nothing	22	2.6
	Total	848	100
What do you do if you experience any side effects while taking medication	I quit medication	226	26.7
	I quit the medicine and start a new one with the same effect	25	2.9
	I consult to a pharmacist	22	2.6
	I consult to a physician	439	51.8
	I consult to my family	42	5
	I do nothing	39	4.6
	Others	46	5.4
	Total	839	98.9
Have you heard the expression of rational drug use and rational use of antibiotics before	Yes	477	56.3
	NO	371	43.8
	Total	848	100

About one-third of students (277) declared that they always read the label on the medication before taking it, and 52% (*n* = 441) of students said they fully understand the contents of drug’s brochure. Also, 40% (343) of students had experienced drugs’ side effects, while 52% (*n* = 439) of students stated that they consult with a doctor in case of drug side effects (Table 2).

**Fig. 2:**
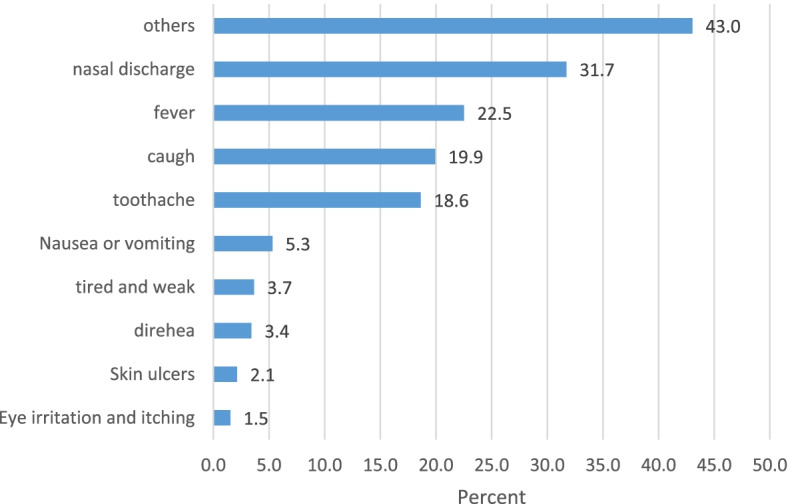
Prevalence of university students with antibiotic self-medication, according to the illness intended to be treated

About two-thirds of students (*n* = 581) had a history of antibiotic use in the past 12 months, and among them 47% (*n* = 400) had used antibiotics without a physician’s prescription (Table 2). The most frequent cases of antibiotic self-medication were related to nasal discharge and fever, which occurred in 32% (*n* = 269) and 23% (*n* = 191) of the students, respectively (Fig. 2).

**Fig. 3:**
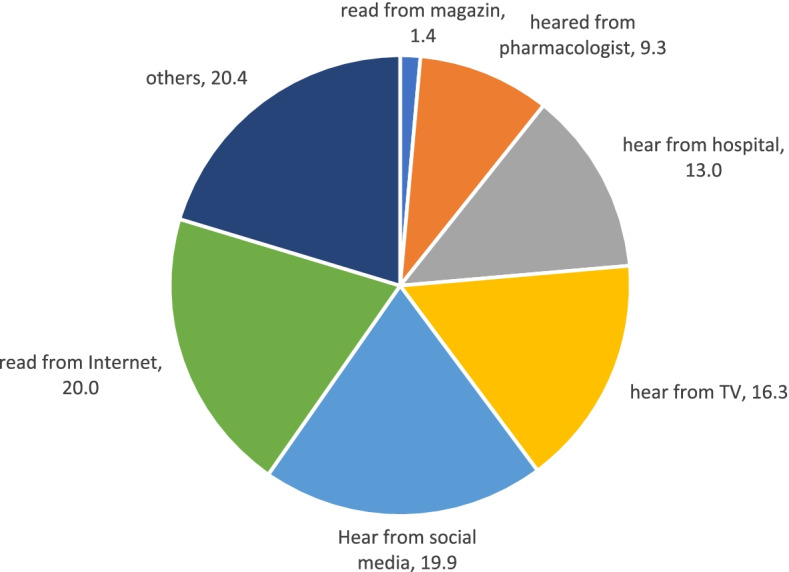
The sources to get the rational drug information among the students of University

Half of the students (*n* = 477) were familiar with the term rational use of drug (Table 2). The internet and social networks were the source of information about the rational use of drug (Fig. 3) in about 40% (*n* = 307) of the students.

There was no significant difference in self-medication between medical and non-medical students (OR = 0.865, 95% CI: 0.659–1.134), (*P* value = 0.3). The prevalence of self-medication in male students was less than female students (OR = 0.76, 95% CI: 0.57–1.00), (P value = 0.6), but this difference was not statistically significant. Self-medication in students with underlying disease was higher than students without underlying disease (OR = 2.8, 95% CI: 1.39–5.60), (*P* value = 0.004). Students who were practicing self-medication were more familiar with the term rational use of drug than others (OR = 1.319, 95% CI: 1.003–1.735), (*P* value = 0.05). The effect of drug use habits on self-medication were as the antibiotic self-medication (OR = 2.63, 95% CI: 1.99–3.48), (P value < 0.001), drug storage at home (OR = 1.58, 95% CI: 1.16–2.13), (P value = 0.003) and the number of unused and unfinished medicines were significantly more prevalent in students with self-medication (OR = 1.33, 95% CI: 1.35–1.57), (P value = 0.05) (Table 3).

**Table 3:** The effect of demographic characteristics and drug use habits on self-medication among university students

		Self-medication		Crude OR (CI95%) ^*^	P value	Adjusted OR (CI95%)	P value
		No N (%)	Yes N (%)				
**University (Medical university/Non-medical university (ref))**		**251 (53.6)**	**190 (50)**	**o.865 (0.659, 1.134)**	**0.3**	**______**	**___**
**sex (Male/Female (ref))**		**261 (68.7)**	**119 (31.3)**	**0.76 (0.57,1.00)**	**0.56**	**______**	**___**
**Economic status (Bad, very bad/Moderate, good, very good (ref))**		**415 (88.7)**	**340 (89.5)**	**1.09 (0.70, 1.67)**	**0.7**	**______**	**___**
**Have chronic disease (yes/No (ref))**		**12 (2.6)**	**26 (6.8)**	**2.8 (1.39, 5.60)**	**0.004**	**2.4 (1.2, 4.9)**	**0.02**
**Reading/checking the instructions in the prospectus (ever/Never or sometimes (ref))**		**166 (35.5)**	**111 (29.2)**	**0.75 (0.56, 1.00)**	**0.54**	**______**	**___**
**Heard the expression of rational drug use and rational use of antibiotics before (yes/No (ref))**		**249 (53.2)**	**228 (60)**	**1.319 (1.003, 1.735)**	**0.047**	**______**	**___**
**Self-medication by antibiotics (yes/No (ref))**		**171 (36.5)**	**229 (60.3)**	**2.63 (1.99, 3.48)**	**< 0.001**	**1.6 (1.2, 2.2)**	**0.002**
**Store drugs (yes/No (ref))**		**107 (22.9)**	**121 (31.8)**	**1.58 (1.16, 2.13)**	**0.003**	**1.5 (1.1, 2.1)**	**0.008**
**The number boxes of drugs they have unused or unfinished in their house**	**None**	**93 (19.9)**	**44 (11.6)**	**1.3 (0.99, 1.66)**	**0.05**	**______**	**___**
	**1–5**	**209 (44.7)**	**136 (35.8)**			**______**	**___**
	**6–10**	**65 (13.9)**	**51 (13.4)**			**______**	**___**
	**> 10**	**101 (21.6)**	**149 (39.2)**			**______**	**___**
Number of boxes of drugs thrown away even without opening the box, since the expiry date has lapsed in the last year	**None**	**190 (40.6)**	**112 (29.5)**	**1.33 (1.35,1.57)**	**< 0.001**	**______**	**___**
	**1–3**	**165 (35.3)**	**135 (35.5)**			**______**	**___**
	**4–7**	**49 (10.5)**	**71 (18.7)**			**______**	**___**
	**8–10**	**26 (5.6)**	**24 (6.3)**			**______**	**___**
	**> 10**	**38 (8.1)**	**38 (10)**			**______**	**___**
**Have experience of complication drug (yes / No (ref))**		**179 (38.2)**	**164 (43.2)**	**1.22 (0.93, 1.62)**	**0.15**	**______**	**___**
**Consulting to a pharmacist if had complication drug (yes/No (ref))**		**10 (2.2)**	**12 (3.2)**	**1.6 (0.54, 4.44)**	**0.41**	**______**	**___**
**Consulting to a physician if had complication drug (yes / No (ref))**		**250 (54)**	**189 (50.3)**	**0.98 (51, 1.89)**	**0.95**	**______**	**___**
**Consulting to family if had complication drug (yes/No (ref))**		**20 (4.3)**	**22 (5.9)**	**1.42 (0.59, 3.42)**	**0.43**	**______**	**___**

^*^ Odds Ratio (Confidence Interval 95%)

The adjusted model showed the students with chronic disease had more self-medication habit than other students OR = 2.4 (95%CI:1.2,4.9, P value =0.02). In addition, the antibiotic self-medication (95%CI:1.2,2.2, P value =0.002) and drug storage at home OR = 1.5 (95%CI:1.1,2.1, P value =0.008) were higher among them than other students in adjusted model (Table 3).

## Discussion

In this study we evaluated the drug use habits and the prevalence of self-medication among university students in Jahrom, Iran. According to the World Health Organization, more than half of the drugs prescribed, distributed and sold worldwide are not clinically justified [20]. Self-medication is an activity in which, medications are usually used by people who may not need them. According to scientific studies in different countries, the prevalence of self-medication varies according to the targeted population [17, 21]. A higher prevalence of self-medication has been reported in developing countries [13]. Students also are among the groups whose self-medication rate is high [14, 22]. The prevalence of self-medication is also higher among medical students and other health-related students [21]. In present study, the prevalence of self-medication practice among students was 44.8%.

In our study, the two most widely used medicines in self-medication were cold remedies and sedatives, which were used by about 33% of the students. However, in addition to these two medications, most studies carried out in Iran, Turkey, Saudi Arabia, Finland and Spain also show that analgesics and antibiotics are also used frequently for self-medication [14, 23, 24]. This result indicates similarity in the use of medications that people use for self-medication. In our study, the prevalence of antibiotic use among students was 47%.

In a study carried out in 2020 in Iran, the prevalence self-medication by antibiotic was 53.3% among nursing students [25]. This rate is higher than in our study. It is thought that the decrease in this number is probably due to the Iran’s policy for antibiotics and its prescription, which has been put in place recently [26]. Self-medication with antibiotics among students, which is more common among medical students, is a topic that many studies have investigated it [27].

Our study showed that 41% of students had one to five packages of unused or unfinished medications at home. Also, about 35% of the students had one to three packages of expired drugs at home per year. This result indicates the typical example of economic burden of self-medication on Iran’s health system. Irrational storage of drugs at home is a global problem. In a systematic review, the prevalence of medicine storage was 35 to 100%, and the wastage of medicine was 17%. This review also stated that southwest region of Asia has the highest rate of drug storage and wastage among urban households [28].

In our study, about one-third of the students reported that they read the instruction of medicines they use. However, 52% of them could fully understand the contents of drug brochure. This number is less than the study conducted in Turkey, which reported over 64% of students were reading the drugs’ instructions [14]. The results of another study indicated that Turkish and Thai students were less likely to notice medication brochures [14, 29].

Social media and cyberspace have been the main source of information for students on self-medication in this study. Putu Eka Arimbawa in a study in Indonesia argued that students’ understanding of drug prescription by physicians has a significant relationship with their understanding of rational use of drug. Use [30]. Our study showed that, students obtain information on medications they use from social media and internet in comparison with television, radio and other source, and this shows the importance of cyberspace in this regard. Providing media-based educational content in universities can be effective in increasing students’ awareness about self-medication.

The results of a study by Tesfaye et al. [31] indicated that medical students in Ethiopia tend to practice self-medication more often than non-medical students, because they have greater knowledge of self-medication. Contrary to the above study, our result identified no significant difference in self-medication between medical and non-medical students in Iran.

Due to the Covid-19 epidemic, one of the limitations of the study was the lack of face-to-face access to students, which in some universities affected student responsiveness. This limitation prolonged the process of collecting data from the university students.

## Conclusions

Self-medication had a high prevalence among university students in Jahrom, Iran. It seems that the best strategy to reduce this high level of self-medication among Iranian students is to raise their awareness and knowledge about the potential risk of self-medication practices through their colleagues. We recommended providing behavioral programs and improving the understanding of drug instructions, especially on appropriate products, to control the self-medication habits of students.

## Data Availability

All data generated or analyzed during this study are included in this published article.
